# Comparison of clinical features of acute lower respiratory tract infections in infants with RSV/HRV infection, and incidences of subsequent wheezing or asthma in childhood

**DOI:** 10.1186/s12879-020-05094-4

**Published:** 2020-05-30

**Authors:** Qin Ding, Lili Xu, Yun Zhu, Baoping Xu, Xiangpeng Chen, Yali Duan, Zhengde Xie, Kunling Shen

**Affiliations:** 1 Beijing Children’s Hospital, Capital Medical University, National Center for Children’s Health, Beijing, 100045 China; 2 Beijing Key Laboratory of Pediatric Respiratory Infection Diseases, Key Laboratory of Major Diseases in Children, Ministry of Education, National Clinical Research Center for Respiratory Diseases, National Key Discipline of Pediatrics (Capital Medical University), Beijing Pediatric Research Institute, Beijing Children’s Hospital, Capital Medical University, National Center for Children’s Health, Beijing, China

**Keywords:** Respiratory syncytial virus, Human rhinovirus, Acute lower respiratory tract infection, Recurrent wheezing, Asthma

## Abstract

**Background:**

To compare the clinical characteristics of acute lower respiratory tract infections (ALRTIs) caused by respiratory syncytial virus (RSV) and human rhinovirus (HRV) and to explore the relationship between the development of recurrent wheezing/asthma and RSV/ HRV infections in infancy.

**Methods:**

Retrospective study was conducted to compare the clinical characteristics of acute lower respiratory tract infections (ALRTIs). Hospitalized patients with ALRTIs from March 2007 to December 2016 were screened. Single RSV cases (s-RSV), single HRV cases (s-HRV), and cases who had co-infection with the two viruses were enrolled. Follow-up was performed to determine whether either specific respiratory virus infection was related to subsequent development of recurrent wheezing/asthma.

**Results:**

The s-RSV children were the youngest (*P* = 0.021), they experienced the most serious condition (*P* < 0.001) and respiratory failure (*P* < 0.001), they also required highest demand of oxygen therapy (*P* < 0.001). And in s-RSV group, the incidence of development of recurrent wheezing was significantly higher in subgroup with the family history of wheezing than that without (*P* < 0.001).

**Conclusion:**

The s-RSV cases suffered from the worst severity of illness, respiratory failure and required highest demand of oxygen therapy. Recurrent wheezing was more common in s-RSV group with family history of wheezing than those without.

## Background

Respiratory syncytial virus (RSV) and human rhinovirus (HRV) are the most common viral pathogens causing respiratory tract infection in infants and young children. RSV is a member of the *Pneumovirus* genus in the *Paramyxoviridae* family. Nearly all children experience at least one RSV infection by the age of 2 years, and in the globle, more than 60% of acute lower respiratory tract infections (ALRTIs) in young children and 80% in infants less than 1 year old are caused by RSV [1]. HRV is a virus belonging to the *Enterovirus* genus in the *Picornaviridae* family. HRV is also a commonly detected virus in nasal/throat swabs from young children who have been hospitalized for wheezing and from infants hospitalized for pneumonia [2, 3]. Although some studies have described the clinical features of RSV and HRV [4, 5], there are few studies about comparison of clinical features of ALRTIs in infants with RSV and HRV infection. The co-infection rate of RSV and HRV is very high in ALRTIs in infants [6], and whether the co-infection is related to the severity of disease also has not been well documented.

The relationship between RSV or HRV ALRTIs in infants with the subsequent development of wheezing/asthma in childhood is still controversial. Some studies found that RSV infection in childhood increased the risk of persistent wheezing and asthma [7, 8]. A rencent Australian study also reported that hospitalization for severe RSV disease in the first 2 years of life was associated with the subsequent hospitalization for the first episode of asthma hospitalisation [9]. Other studies found that wheezing caused by HRV in infants and young children increased the risk of childhood asthma [10] and diagnosis of childhood asthma as well as acute asthma attacks were also related to HRV infection [11, 12]. A research from Finland reported that current asthma was present in 64% of the cases with HRV-induced wheezing [13]. Kumar also found that about 50–85% of acute asthma exacerbations were caused by viral infections, of which 2/3 were caused by HRV [14].

As mentioned above, the co-infection rate of RSV and HRV was very high in ALRTIs in infants [6]. Whether the co-infection of RSV and HRV ALRTIs in infants has an impact on the subsequent development of wheezing/asthma in childhood was not clear. So, in this study, we compared the clinical characteristics of ALRTIs caused by single RSV infection (s-RSV), single HRV infection (s-HRV) and co-infection with these two viruses (co-infection) in infants, and explored whether the incidences of subsequent wheezing/asthma in childhood after ALRTIs with s-RSV, s-HRV and co-infection in infancy were different.

## Methods

### Patients

From March 2007 to December 2016, children who were diagnosed with ALRTIs and hospitalized at Beijing Children’s Hospital were screened for this study. ALRTIs were defined by the presence of signs and symptoms of respiratory tract infection (i.e., fever, coughing, tachypnea, dyspnea, wheezing or rales on auscultation) [15]. The inclusion criteria of patients are as followes: (1) less than 3 years of age; (2) the infectious agent was single RSV, single HRV, or co-infection with both. Patients with any of the following conditions were excluded: (1) immune deficiency; (2) congenital heart disease; (3) congenital airway and lung malformations or dysplasia; (4) foreign bodies in the airway; (5) co-infection with other pathogens; (6) sepsis, tumor, or leukemia; and (7) trauma.

### Collection of specimens and clinical information

Nasopharyngeal aspirates or throat swab specimens were collected from each patient within 24 h of hospitalization. All samples were stored at − 80 °C until testing.

Age, gender, symptoms, signs, exam results, diagnoses, complications, the need for oxygen and family history of wheezing were examined (Table 1), and all cases were scored according to their clinical characteristics (Supplement 1) [16] The diagnostic criteria for respiratory failure are as follows [17]: PaO_2_ < (arterial blood) with or without PaCO_2_ > 45 mmHg. Heart failure would be diagnosed if meeting 4 of the following 6 criteria: (1) respiratory rate accelerated suddenly (> 60 beats/min); (2) heart rate elevated suddenly (> 180 beats/min); (3) dysphoria and cyanosis; (4) gallop rhythm, jugular venous distention and muffled heart sounds; (5) liver enlargement; (6) oliguria or anuria, and edema. Increase of creatine kinase isoenzyme (CK-MB) > 25 U/L or/and changing of ST-T in electrocardiogram (ECG) indicate myocardial damage. Aspartate aminotransferase (AST) > 40 U/L or/and alanine aminotransferase (ALT) > 40 U/L reflect liver dysfunction.

**Table 1:** Clinical features of ALRTIs with different viruses

	s-RSV (*n* = 199)	s-HRV (*n* = 47)	Co-infection (*n* = 73)	*P*
Age (yr)($\overline{X}\pm S$)	0.46 ± 0.56^d^	0.80 ± 0.78^d^	0.53 ± 0.47	0.021^*^
Gender				
M *n*(%)	137 (68.84)	38 (80.85)	56 (76.71)	0.164
F *n*(%)	62 (31.16)	9 (19.15)	17 (23.29)	
Temperature (°C)($\overline{X}\pm S$)	37.94 ± 0.75	38.10 ± 0.85	37.55 ± 4.52	0.349
Wheezing *n*(%)	112 (56.28)	28 (59.57)	47 (64.38)	0.480
Lung infiltrates *n*(%)	134 (67.34)	35 (74.47)	47 (64.38)	0.506
WBC(*10^9^) ($\overline{X}\pm S$)	9.59 ± 5.04	9.89 ± 5.11	9.44 ± 4.60	0.889
PMN(*10^9^) ($\overline{X}\pm S$)	35.73 ± 15.38	38.50 ± 17.96	35.16 ± 18.54	0.526
EOS(*10^9^) ($\overline{X}\pm S$)	0.26 ± 0.30	0.25 ± 0.20	0.30 ± 0.46	0.561
CRP^b^	31/195^c^	5/47	4/71^c^	0.076
Complications				
Liver damage *n*(%)	38 (19.10)	5 (10.64)	11 (15.07)	0.338
Myocardial damage *n*(%)	69 (34.67)	11 (23.40)	29 (39.73)	0.179
Respiratory failure *n*(%)	50 (25.13)	5 (10.64)	5 (6.85)	< 0.001^*^
Heart failure *n*(%)	25 (12.63)	1 (2.13)	8 (10.96)	0.111
Oxygen therapy^a^*n*(%)	148 (74.37)	22 (46.81)	50 (68.49)	0.001^*^
NCPAP *n*(%)	50 (25.13)	5 (10.64)	6 (8.22)	0.002^*^
Clinical score^e^ ($\overline{X}\pm S$)	4.10 ± 2.32^d^	3.13 ± 1.65^d^	3.25 ± 1.47^d^	< 0.001^*^
Bronchiolitis n(%)	39 (19.60)	4 (8.51)	18 (24.66)	0.086
Pneumonia n(%)	155 (77.89)	40 (85.11)	53 (72.60)	0.274
Bronchitis n(%)	5 (2.51)	3 (6.38)	2 (2.74)	0.365

^a^Oxygen therapy includes nasal catheter oxygen inhalation and nasal continuous positive airway pressure (NCPAP);

^b^CRP (C-Reactive Protein): Only when the value of CRP was greater than/ equal to 8, the specific value would be displayed;

^c^In s-RSV group, CR*P* values were collected from 195 patients, and there were 31 patients with CRP greater than 8; all the 47 cases in s-HRV group were tested for CRP, and 5 cases with CRP greater than 8; in co-infection group, CRP values were collected from 71 patients, and only 4 patients with CRP greater than 8;

^d^Games-Howell test; ^e^ The clinical score system was showed in supplement 1

*P value of < 0.05 was considered statistically significant

### Preparation of nucleic acids

Total nucleic acids (DNA and RNA) were extracted from 200 μl nasopharyngeal aspirates or throat swab specimens using the Nucli Senseasy MAG™ automated extraction system (bioMérieux, Marcyl’ Etoile, France) according to the manufacturer’s instructions and eluted with 60 μl elution buffer.

### Detection of respiratory viruses

The presence of common respiratory viral agents, including RSV, parainfluenza virus (PIV) type 1–4, 2009 H1N1 influenza virus (2009H1N1), H3 subtype influenza virus(H3), seasonal H1 subtype influenza virus(H1), influenza B virus (Flu B), human rhinovirus/enterovirus (HRV/HEV), human coronavirus (HCoV 229E, NL63, HKU1, and OC43), human metapneumovirus (HMPV), human bocavirus (HBoV), and human adenovirus (HAdV), were determined using multiplex RT-PCR, single RT-PCR, or PCR assays as described previously [18, 19]. Blank virus transport media served as a negative control for nucleic acid extraction and PCR analysis.

### Follow up

Telephone follow-up was conducted to assess the subsequent development of wheezing episode and asthma in childhood at June, 2017. The questionnaire of follow-up is shown in the supplementary materials (Supplement 2).

### Statistical analysis

Continuous variables are expressed as means ± standard deviations (SD) or medians. For categorical variables, the relative numbers of patients in each category were calculated. Differences between groups were assessed using Pearson’s Chi square test or Fisher’s exact test for categorical variables and one-way analysis of variance (ANOVA) and independent-samples test for continuous variables. Games-Howell test was used for the data that do not meet the hypothesis of variance homogeneity. All analyses were performed using SPSS software, version 20.0 (IBM Corporation, Armonk, NY, USA). All calculations were made in a two-tailed manner, and *P* value < 0.05 was considered statistically significant.

## Results

### Patients enrolled

**Fig. 1:**
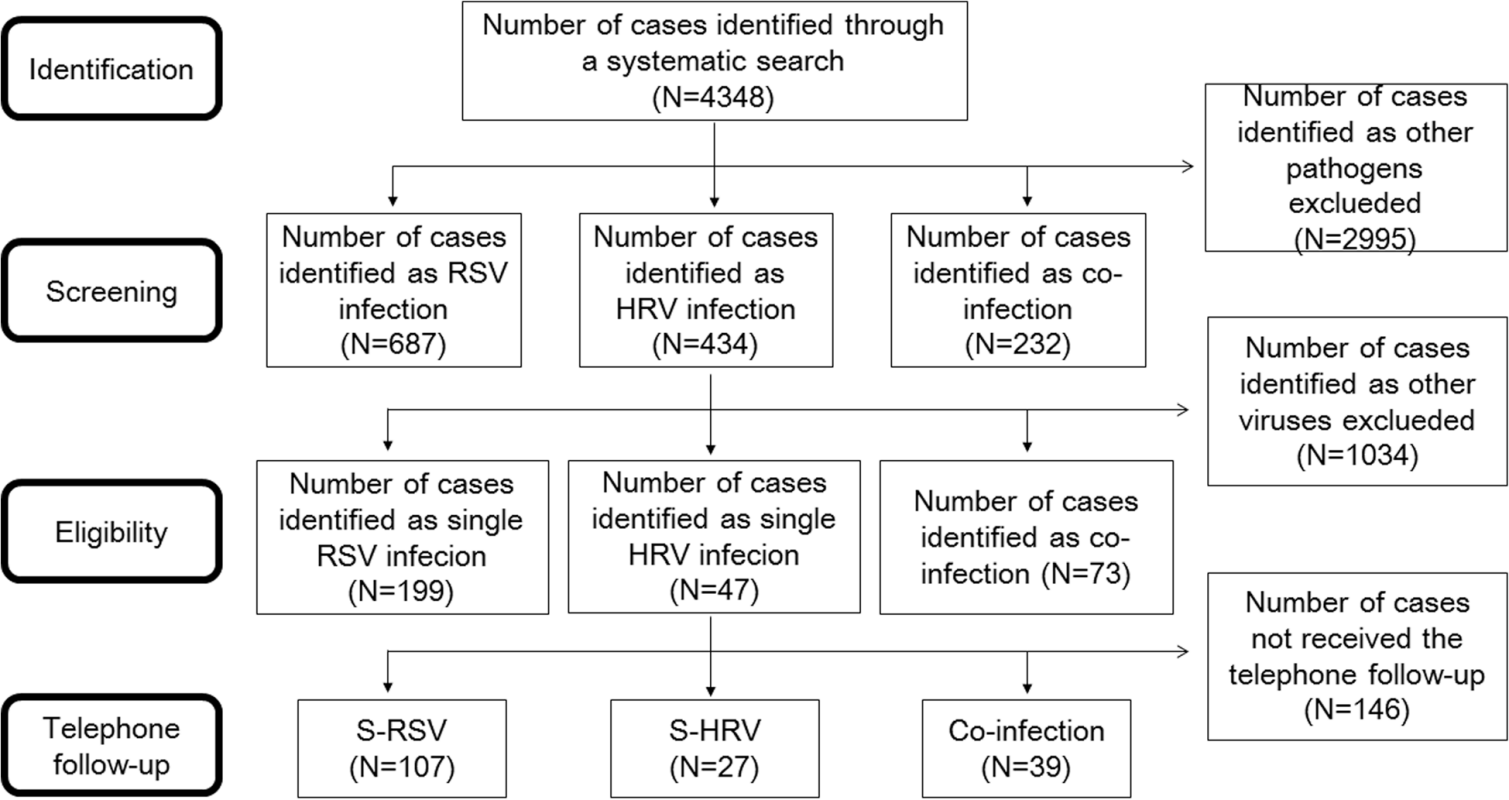
The process of case screening

**Fig. 2:**
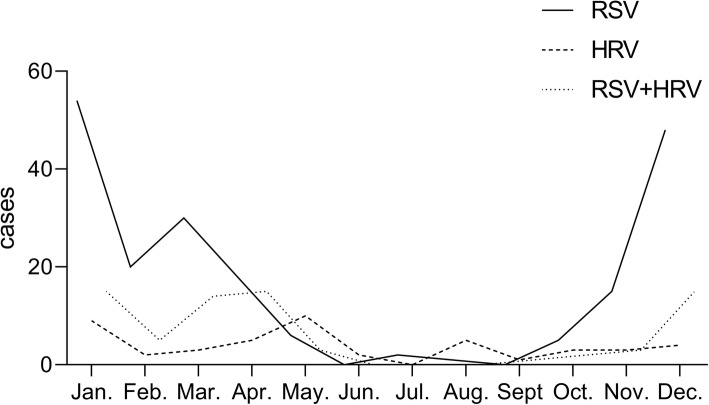
The seasonal distribution of cases

**Fig. 3:**
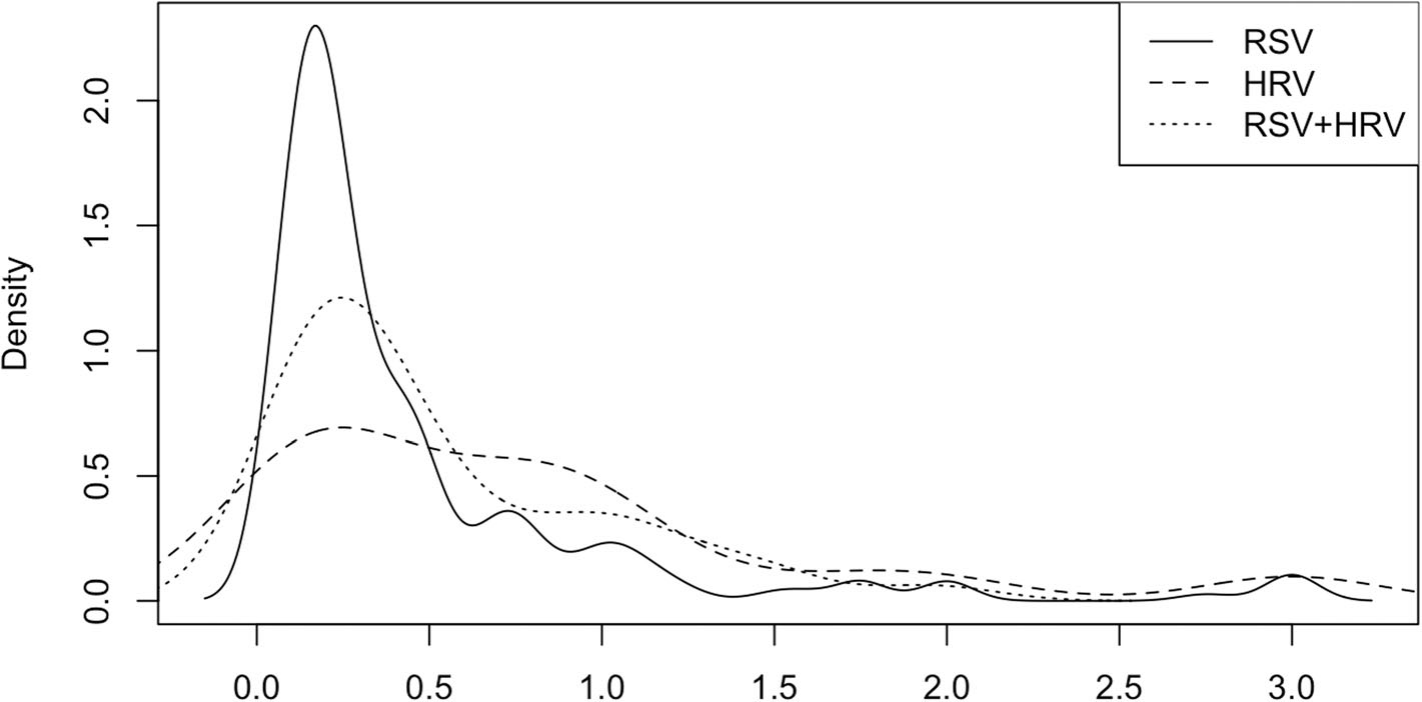
The distribution of age(year)

From March 2007 to December 2016, a total of 4348 children were screened for this study, and 319 children were finally enrolled, including 199 s-RSV, 47 s-HRV and 73 co-infection cases through the selection process (Fig. 1). January was the peak month for these three groups. March and December were the peak months for s-RSV and co-infection. (Fig. 2). The mean age was 0.46 ± 0.56 years, 0.80 ± 0.78 years and 0.53 ± 0.47 years in s-RSV group, s-HRV group and co-infection group respectively, and most of them were less than 1 year (Fig. 3). The male-to-female ratio of was 2.21:1, 4.22:1 and 3.29:1 in s-RSV group, s-HRV group and co-infection group respectively.

### Clinical features and main diagnoses of the enrolled cases

The mean age of s-RSV group was younger than that of s-HRV group (*P* = 0.021, Table 1). There were significant differences in disease scores among the three groups (*P* < 0.001, Table 1), with the average disease scores showing the following trend: s-RSV group> co-infection group > s-HRV group. This result indicated that s-RSV group had more severe disease than s-HRV or co-infection groups.

The s-RSV group was most likely to experience respiratory failure (*P* < 0.001, Table 1). And both the oxygen therapy (nasal catheter oxygen inhalation and nasal continuous positive airway pressure, NCPAP) (*P* = 0.001, Table 1) and NCPAP (*P* = 0.001, Table 1) were highest in s-RSV group.

There were no statistically significant differences among the three groups in terms of gender, temperature, wheezing, lung infiltrates, the count of WBC, PMN, eosinophil, CRP, liver damage, myocardial damage, or heart failure during hospitalization.

Upon discharge there were no significant differences in clinical diagnoses of bronchiolitis, pneumonia, and bronchitis among the three groups (Table 1). Moreover, when stratified by family history, there were no statistically significant differences in the major clinical diagnosis (Table 2).

**Table 2:** The main clinical diagnosis in groups with/without family history of wheezing(%)

		Bronchiolitis *n*(%)	Pneumonia *n*(%)	Bronchitis *n*(%)
P (*n* = 72)	s-RSV (*n* = 42)	12 (28.57)	29 (69.05)	1 (2.38)
	s-HRV (*n* = 11)	1 (9.09)	9 (81.82)	1 (9.09)
	Co-infection (*n* = 19)	5 (26.32)	13 (68.42)	1 (5.26)
	χ2	1.624	0.761	1.794^□^
	*P*	0.512	0.684	0.374
N (*n* = 101)	s-RSV (*n* = 65)	9 (13.85)	54 (83.08)	2 (3.08)
	s-HRV (*n* = 16)	1 (6.25)	14 (87.50)	1 (6.25)
	Co-infection (*n* = 20)	1 (5.00)	18 (90.00)	1 (5.00)
	χ2	1.148^□^	0.492^□^	1.163^□^
	*P*	0.576	0.918	0.449
Total (*n* = 173)	s-RSV (*n* = 107)	21 (19.63)	83 (77.57)	3 (2.80)
	s-HRV (*n* = 27)	2 (7.41)	23 (85.19)	2 (7.41)
	Co-infection (*n* = 39)	6 (15.38)	31 (79.49)	2 (5.13)
	χ2	2.375	0.761	1.858^□^
	*P*	0.305	0.683	0.465

□Fisher’s exact test

P: positive family history of wheezing. N: negative family history of wheezing

### Results of follow-up

A total of 173 cases, including 107 s-RSV cases, 27 s-HRV cases and 39 co-infection cases, received a telephone follow-up 6 months to 10.3 years after discharge. The rate of recurrent wheezing of the three group was 41.1% (s-RSV), 22.2% (s-HRV) and 41% (co-infection), while the rate of asthma was 7.5% (s-RSV), 11.1% (s-HRV) and 12.8% (co-infection). But whether the family history was positive or not, there was no significant difference in recurrent wheezing/asthma among the three groups (Table 3).

**Table 3:** Incidence of recurrent wheezing/asthma in groups with/without family history of wheezing(%)

Family history	Groups	Recurrent wheezing N (%)	Asthma N (%)
P (*n* = 72)	s-RSV(*n* = 42)	28 (66.67)	3 (7.14)
	s-HRV(*n* = 11)	3 (27.27)	3 (27.27)
	co-infection(*n* = 19)	11 (57.89)	4 (21.05)
	*P*	0.062	0.120^□^
N (*n* = 101)	s-RSV(*n* = 65)	16 (24.62)	5 (7.69)
	s-HRV(*n* = 16)	3 (18.75)	0 (0.00)
	co-infection(*n* = 20)	5 (25.00)	1 (5.00)
	*P*	0.889^□^	0.829^□^

□Fisher’s exact test

P: positive family history of wheezing. N: negative family history of wheezing

Each group was further classified into two subgroups by family history of wheezing. Only in s-RSV group, the incidence of recurrent wheezing was significantly higher in the subgroup with family history of wheezing than the subgroup without family history of wheezing (*P* < 0.001, Table 4), but the incidence of asthma was not significantly different between these two subgroups (*P* = 1.000, Table 4). In the other two groups, no significant differences in recurrent wheezing/asthma were found between subgroups (*P* > 0.05, Table 4). This result indicated that patients with family history of wheezing were more likely to develop recurrent wheezing after ALRTIs caused by RSV in infancy than those without.

**Table 4:** Incidence of recurrent wheezing/asthma in subgroups with/without family history of wheezing

Groups	Family history of wheezing	Recurrent wheezing N(%)	Asthma N(%)
s-RSV(*n* = 107)	P(*n* = 42)	28 (66.67)	3 (7.14)
	N(*n* = 65)	16 (24.62)	5 (7.69)
	*P*	< 0.001	1.000^□^
s-HRV(*n* = 27)	P(*n* = 11)	3 (27.27)	3 (27.27)
	N(*n* = 16)	3 (18.75)	0 (0.00)
	*P*	0.662	0.056^□^
Co-infection(*n* = 39)	P (*n* = 19)	11 (57.89)	4 (21.05)
	N(*n* = 20)	5 (25.00)	1 (5.00)
	*P*	0.054^□^	0.182^□^

□Fisher’s exact test

P: positive family history of wheezing. N: negative family history of wheezing

## Discussion

### Differences in clinical manifestations of ALRTIs with s-RSV, s-HRV, and co-infection

In infants and young children, ALRTIs are most frequently caused by respiratory viruses, such as RSV and HRV, both of which play a significant role in pediatric respiratory tract infections [20, 21]. Co-infection is common in ALRTIs, while RSV and HRV are the most common viruses in co-infections, with a rate higher than 50% [22]. Our findings provide details on the characteristics of s-RSV, s-HRV and co-infection of the two viruses among children under 3 years of age in Beijing Children’s Hospital. Our study showed that the peak months for s-HRV and s-HRV infection were January. And the mean age of children in s-HRV group was older than those in the other groups, but most cases in every group were less than 1 year. The s-RSV cases were more likely to experience the respiratory failure and had the highest demand of nasal catheter oxygen inhalation and NCPAP. These results were consistent with the disease score and indicated that s-RSV cases was more severe than co-infection groups. Another study also suggested that RSV infection was more likely to cause coughing, wheezing, and shortness of breath than HRV infection [23].

It’s controversial about the role of co-infection of viruses in the severity of disease. Martin et al. found that comparing with children infected with a single virus, children with mixed viruses infection were less frequently admitted to the inpatient ward or to the intensive care unit and less likely to require supplemental oxygen or hospital stays longer than 3 days [24]. Nascimento et al. found that co-infections were not associated with poorer outcomes [25]. A prospective analysis including 322 hospitalized infant patients with acute respiratory disease (ARD) revealed that co-infections were significantly more common in mild respiratory diseases [26]. Brand et al. also found that the severity of illness in children with bronchiolitis is not associated with multiple viruses infection [27]. These results are coincident with that of our study, and demonstrated that co-infection in ALRTIs could not cause more severe condition. A most recent Italian research showed that infants with co-infection seemed to mount a lower inflammatory response [28], the antagonism caused by non-specific innate immune responses stimulated by co-infection virus maybe a possible reason.

However, other studies had different results. Calvo et al. reported that patients with mixed viruses infection had more frequent fever, longer hospital stays, and more antibiotic treatment than patients infected with RSV alone [29]. Aberle et al. also reported that co-infection (HRV and HAdV) with RSV was associated with the severity of illness, hypoxemia, and fever [30]. Richard et al. found that dual viral infection was a relevant risk factor for admission to PICU [31]. The different results of co-infection of viruses on the severity of disease may be associated with several reasons, such as study population, research design, and standard of evaluation and so on. Multicenter studies are needed.

### Incidences of subsequent wheezing/asthma in childhood

A large number of studies have identified a link between RSV/HRV infection and an increased likelihood to develop either recurrent wheezing or asthma. Previous studies showed that with RSV infection, the risk of developing recurrent wheezing was from 28.1 to 30% [32, 33], and for asthma, the rate was from 30 to 38.4% [32, 34]. For HRV-infected children, the risk for developing asthma at 6 years of age being more than four-fold higher compared with HRV-negative cases [35]. In the large, prospective Childhood Origins of Asthma (COAST) study performed in children at increased risk of developing allergies and asthma, HRV infections represented a significantly increased risk (OR = 10) for developing asthma by 6 years of age [10]. From our study, the rate of recurrent wheezing of the three group was 41.1% (s-RSV), 22.2% (s-HRV) and 41% (co-infection), while the rate of asthma was 7.5% (s-RSV), 11.1% (s-HRV) and 12.8% (co-infection). Taking the number of enrolled cases into account, especially s-HRV and co-infection cases were small in our study and this may also a potential bias of the result. So, further studies are needed to explore the mechanism of different immune response to RSV and HRV infection in infants.

We found that there were no significant differences in the development of asthma among subgroups of the three groups with or without family history of wheezing. And only s-RSV group with positive family history had a tend to have recurrent wheezing than those without. This result is consistent with that of the following studies. A Japanese study found no connection between RSV-based ALRTIs in infants and childhood asthma [36]. Kusel, et al. reported that RSV and HRV infections in the first year after birth were associated with wheezing and asthma at the age of 5 years, with no difference between the two groups [37]. Uppala R.et al. reported that the specific pathogens (RSV/HRV) did not account for a statistically significant difference in subsequent wheezing or asthma development [38].

However, some studies supported that RSV or HRV infection in early childhood was associated with chronic airway diseases, especially in the subsequent development of wheezing and asthma. From some population-based birth cohort studies, RSV bronchiolitis was found to be associated with subsequent wheezing and asthma, especially in children with family histories of atopy and asthma [32, 39]. Another high-risk birth cohort study showed that among outpatients viral wheezing illnesses in infancy and early childhood, those associated with HRV infections were the most significant risk factors of subsequent development of asthma at the age of 6 years [10]. We found the different immune response induced by RSV and HRV may be the cause of this result. For instance, IL-8 level in the nasal aspirate is higher in ALRTIs associated with RSV in children than those associated with HRV, and higher respiratory tract IL-8 level was associated with hypoxia and need for ventilation in infants [40, 41]. Another previous research reported that RSV bronchiolitis, with higher nasal levels of IFN-λ than HRV infection, and higher nasal IFN-λ levels were associated with increased disease severity [42, 43], and severe bronchiolitis was the risk factor for recurrent wheezing [44]. Other studies suggested that IL-5 was significantly elevated in the HRV group compared with the RSV group in both serum and nasal secretions [45], and as we all know, IL-5 has the effect of inducing eosinophil accumulation in airways.

## Conclusions

The severity of disease in s-RSV cases was higher than that of s-HRV or Co-infection groups, with a higher demand for nasal catheter oxygen inhalation and NCPAP and higher risk for respiratory failure. No matter with or without family history of wheezing, there are no significant differences in the incidence of subsequent development of recurrent wheezing and asthma among s-RSV, s-HRV and co-infection groups. But patients with family history of wheezing are subject to have recurrent wheezing after ALRTIs caused by RSV in infancy than those without the family history.

## Supplementary information

**Additional file 1: Supplement 1.** Clinical Scoring System. **Supplement 2.** Follow-up questionnaire for recurrent wheezing and asthma.

## Data Availability

The datasets supporting the conclusions of this article are included within the article.

## Abbreviations

RSV: Respiratory syncytial virus
HRV: Human rhinovirus
ALRTIs: Acute lower respiratory tract infections
s-RSV: Single RSV infection
s-HRV: Single HRV infection
WBC: White blood cell
PMN: Polymorphonuclear neutrophil
CRP: C-Reactive Protein

## References

[1] Wright M, Piedimonte G (2011). Respiratory syncytial virus prevention and therapy. Past, present, and future. Pediatr Pulmonol.

[2] Miller EK, KhuriBulos N, Williams JV, Shehabi AA, Faouri S, Jundi IA (2009). Human rhinovirus C associated with wheezing in hospitalized children in the Middle East. J Clin Virol.

[3] Khetsuriani N, Kazerouni NN, Erdman DD, Lu X, Redd SC, Anderson LJ (2007). Prevalence of viral respiratory tract infections in children with asthma. J Allergy Clin Immunol.

[4] Hall CB, Simőes EA, Anderson LJ (2013). Clinical and epidemiologic features of respiratory syncytial virus. Curr Top Microbiol Immunol.

[5] Tran DN, Trinh QD, Pham NT, Pham TM, Ha MT, Nguyen TQ (2016). Human rhinovirus infections in hospitalized children. Clinical, epidemiological and virological features. Epidemiol Infect.

[6] Xie ZD, Xiao Y, Liu CY, Hu YH, Yao Y, Yang Y (2011). Three years surveillance of viral etiology of acute lower respiratory tract infection in children from 2007 to 2010. Zhonghua Er Ke Za Zhi.

[7] Sala KA, Moore A, Desai S, Welch K, Bhandari S, Carroll CL (2015). Factors associated with disease severity in children with bronchiolitis. J Asthma.

[8] Homaira N, Oei JL, Mallitt KA, Abdel-Latif ME, Hilder L, Bajuk B (2016). High burden of RSV hospitalization in very young children. A data linkage study. Epidemiol Infect.

[9] Homaira N, Briggs N, Pardy C, Hanly M, Oei JL, Hilder L (2017). Association between respiratory syncytial viral disease and the subsequent risk of the first episode of severe asthma in different subgroups of high-risk Australian children. a whole-of-population-based cohort study. BMJ Open.

[10] Jackson DJ, Gangnon RE, Evans MD, Roberg KA, Anderson EL, Pappas TE (2008). Wheezing rhinovirus illnesses in early life predict asthma development in high-risk children. Am J Respir Crit Care Med.

[11] Zheng XY, Xu YJ, Guan WJ, Lin LF (2018). Regional, age and respiratory-secretion-specific prevalence of respiratory viruses associated with asthma exacerbation. A literature review. Arch Virol.

[12] Abraham AM, Baraket A, Bialasiewicz S, Caniza MA, Chan PK, Cohen C (2017). Global epidemiology of non-influenza RNA respiratory viruses. Data gaps and a growing need for surveillance. Lancet Infect Dis.

[13] Backman K, Ollikainen H, Piipposavolainen E, Nuolivirta K, Korppi M (2018). Asthma and lung function in adulthood after a viral wheezing episode in early childhood. Clin Exp Allergy.

[14] Kumar RK, Foster PS, Rosenberg HF (2014). Respiratory viral infection, epithelial cytokines, and innate lymphoid cells in asthma exacerbations. J Leukoc Biol.

[15] Liu C, Xiao Y, Zhang J, Ren L, Li J, Xie Z (2015). Adenovirus infection in children with acute lower respiratory tract infections in Beijing, China, 2007 to 2012. BMC Infect Dis.

[16] Larrañaga CL, Ampuero SL, Luchsinger VF, Carrión FA, Aguilar NV, Morales PR (2009). Impaired immune response in severe human lower tract respiratory infection by respiratory syncytial virus. Pediatr Infect Dis J.

[17] Jiang ZF, Hu YM (2015). Zhu Futang practice of pediatrics. People’s Medical Publishing House.

[18] Ren L, Gonzalez R, Wang Z, Xiang Z, Wang Y, Zhou H (2009). Prevalence of human respiratory viruses in adults with acute respiratory tract infections in Beijing, 2005–2007. Clin Microbiol Infect.

[19] Guo L, Gonzalez R, Xie Z, Zhou H, Liu C, Wu C (2011). Bocavirus in children with respiratory tract infections. Emerg Infect Dis.

[20] Hall CB, Weinberg GA, Iwane MK, Blumkin AK, Edwards KM, Staat MA (2009). The burden of respiratory syncytial virus infection in young children. N Engl J Med.

[21] Manoha C, Espinosa S, Aho SL, Huet F, Pothier P (2007). Epidemiological and clinical features of hMPV, RSV and RVs infections in young children. J Clin Viro.

[22] Jun L (2015). Chunyan L, Zhengde X, lili R, Baoping X, Jianwei W, et al. study of single infections and mixed infections by respiratory syncytial virus in children with acute lower respiratory tract disease. Chinese J Pract Pediatr.

[23] Sun Q, Chen Z, Huang L, Zhu C, Wang Y, Wang M (2014). A comparison of clinical features between rhinovirus and respiratory syncytial virus infection in lower respiratory tract in children. J Clin Pediatr.

[24] Martin ET, Kuypers J, Wald A, Englund JA (2012). Multiple versus single virus respiratory infections. Viral load and clinical disease severity in hospitalized children. Influenza Other Respir Viruses.

[25] Nascimento MS, Souza AV, Ferreira AV, Rodrigues JC, Abramovici S, Silva Filho LV (2010). High rate of viral identification and coinfections in infants with acute bronchiolitis. Clinics..

[26] Canducci F, Debiaggi M, Sampaolo M, Marinozzi MC, Berrè S, Terulla C (2008). Two-year prospective study of single infections and co-infections by respiratory syncytial virus and viruses identified recently in infants with acute respiratory disease. J Med Virol.

[27] Brand HK, De GR, Galama JM, Brouwer ML, Teuwen K, Hermans PW (2012). Infection with multiple viruses is not associated with increased disease severity in children with bronchiolitis. Pediatr Pulmonol.

[28] Petrarca L, Nenna R, Frassanito A, Pierangeli A, Leonardi S, Scagnolari C (2018). Acute bronchiolitis. Influence of viral co-infection in infants hospitalized over 12 consecutive epidemic seasons. J Med Virol.

[29] Calvo C, García-García ML, Blanco C, Vázquez MC, Frías ME, Pérez-Breña P (2008). Multiple simultaneous viral infections in infants with acute respiratory tract infections in Spain. J Clin Virol.

[30] Aberle JH, Aberle SW, Pracher E, Hutter HP, Kundi M, Popow-Kraupp T (2005). Single versus dual respiratory virus infections in hospitalized infants. impact on clinical course of disease and interferon-gamma response. Pediatr Infect Dis J.

[31] Richard N, Komurianpradel F, Javouhey E, Perret M, Rajoharison A, Bagnaud A (2008). The impact of dual viral infection in infants admitted to a pediatric intensive care unit associated with severe bronchiolitis. Pediatr Infect Dis J.

[32] Henderson J, Hilliard TN, Sherriff A, Stalker D, Al Shammari N, Thomas HM (2005). Hospitalization for RSV bronchiolitis before 12 months of age and subsequent asthma, atopy and wheeze. A longitudinal birth cohort study. Pediatr Allergy Immunol.

[33] Sigurs N, Gustafsson PM, Bjarnason R, Lundberg F, Schmidt S, Sigurbergsson F (2005). Severe respiratory syncytial virus bronchiolitis in infancy and asthma and allergy at age 13. Am J Respir Crit Care Med.

[34] Sigurs N, Bjarnason R, Sigurbergsson F, Kjellman B (2000). Respiratory syncytial virus bronchiolitis in infancy is an important risk factor for asthma and allergy at age 7. Am J Respir Crit Care Med.

[35] Kotaniemi-Syrjanen A, Vainionpaa R, Reijonen TM, Waris M, Korhonen K, Korppi M (2003). Rhinovirus-induced wheezing in infancy--the first sign of childhood asthma?. J Allergy Clin Immunol.

[36] Narita A, Nishimura N, Arakawa Y, Suzuki M, Sakamoto K, Sakamoto M (2011). Relationship between lower respiratory tract infections caused by respiratory syncytial virus and subsequent development of asthma in Japanese children. Jpn J Infect Dis.

[37] Kusel MM, de Klerk NH, Kebadze T, Vohma V, Holt PG, Johnston SL (2007). Early-life respiratory viral infections, atopic sensitization, and risk of subsequent development of persistent asthma. J Allergy Clin Immunol.

[38] Teeratakulpisarn J, Pientong C, Ekalaksananan T, Ruangsiripiyakul H, Uppala R (2014). Rhinovirus infection in children hospitalized with acute bronchiolitis and its impact on subsequent wheezing or asthma. a comparison of etiologies. Asian Pac J Allergy Immunol.

[39] Sigurs N, Bjarnason R, Sigurbergsson F, Kjellman B, Björkstén B (1995). Asthma and immunoglobulin E antibodies after respiratory syncytial virus bronchiolitis. A prospective cohort study with matched controls. Pediatrics..

[40] Warris A, Neeleman C, Staal FJT, Preijers F, Ferwerda G, Brand HK (2013). CD4+ T-cell counts and interleukin-8 and CCL-5 plasma concentrations discriminate disease severity in children with RSV infection. Pediatr Res.

[41] Díaz PV, Valdivia G, Gaggero AA, Bono MR, Zepeda G, Rivas M (2015). Pro-inflammatory cytokines in nasopharyngeal aspirate from hospitalized children with respiratory syncytial virus infection with or without rhinovirus bronchiolitis, and use of the cytokines as predictors of illness severity. Medicine..

[42] Selvaggi C, Pierangeli A, Fabiani M, Spano L, Nicolai A, Papoff P (2014). Interferon lambda 1-3 expression in infants hospitalized for RSV or HRV associated bronchiolitis. J Inf Secur.

[43] Nenna R, Ferrara M, Nicolai A, Pierangeli A, Scagnolari C, Papoff P (2015). Viral load in infants hospitalized for respiratory syncytial virus bronchiolitis correlates with recurrent wheezing at thirty-six-month follow-up. Pediatr Infect Dis J.

[44] Bai J (2014). Study on related factors of bronchiolitis and subsequent recurrent wheezing in infants. Maternal Child Health Care China.

[45] Kato M, Tsukagoshi H, Yoshizumi M, Saitoh M, Kozawa K, Yamada Y (2011). Different cytokine profile and eosinophil activation are involved in rhinovirus- and RS virus-induced acute exacerbation of childhood wheezing. Pediatr Allergy Immunol.

