# Supplementary material for: Comparison of clinical features of acute lower respiratory tract infections in infants with RSV/HRV infection, and incidences of subsequent wheezing or asthma in childhood
Source: BMC Infect Dis. 2020 May 30;20:387. doi: 10.1186/s12879-020-05094-4 (PMC7260463; doi:10.1186/s12879-020-05094-4)
Supplement: Supplementary file 1 — Additional file 1: Supplement 1. Clinical Scoring System. Supplement 2. Follow-up questionnaire for recurrent wheezing and asthma. [file 12879_2020_5094_MOESM1_ESM.zip › Supplement 1 Clinical Scoring System_ESM.docx]

Supplement 1. Clinical Scoring System

| factors | Score | | | | Maximal Score per Item |
| --- | --- | --- | --- | --- | --- |
|  | 0 | 1 | 2 | 3 |  |
| Hospitalization | No | ≤5d | ＞5d | CCU† | 0-5 |
| Supplemental oxygen | No | Yes | ＜3d | ＞3d | 0-4 |
| Maximal FiO2 (%) | 21 | 22-30 | ≥31 | MV‡+ | 0-5 |
| Maximal score value | 0 | 1-3 | 2-7 | 4-14 | 0-14 |

* Score: 0 to 3 indicates mild disease, 4 to 6 indicates moderate disease, and 7 to 14 indicates severe disease.

† CCU indicates critical care unit.

‡Mechanical ventilation. Supplemental oxygen and increasing concentrations of FiO2 were administred when pulse oximetric reading was less than 95% at room air.
