# Supplementary material for: Comparison of clinical features of acute lower respiratory tract infections in infants with RSV/HRV infection, and incidences of subsequent wheezing or asthma in childhood
Source: BMC Infect Dis. 2020 May 30;20:387. doi: 10.1186/s12879-020-05094-4 (PMC7260463; doi:10.1186/s12879-020-05094-4)
Supplement: Supplementary file 1 — Additional file 1: Supplement 1. Clinical Scoring System. Supplement 2. Follow-up questionnaire for recurrent wheezing and asthma. [file 12879_2020_5094_MOESM1_ESM.zip › Supplement 2 Follow-up questionnaire for recurrent wheezing and asthma_ESM.docx]

Supplement 2 Follow-up questionnaire for recurrent wheezing and asthma

ID. Gender Age

1. Did your child have a wheezing after he or she was discharged from the hospital?

A. Yes B. No

2. Is there a cause of your child's wheezing attack?

A. Yes B. No

3. If there is, what is the cause of your child's wheezing attack?

A. Respiratory infection B. Contact allergens

4. How many times of wheezing episodes did your child have per year?

A. ＜3 wheezing episodes/year B. ≥3 wheezing episodes/year

5. Was your child diagnosed with asthma by a pediatrician?

A. Yes B. No

6. Any other special event or emergencies about wheezing or asthma occurred?
